# A Pilot Rapid Triage Process for Prehospital ST-Segment Myocardial Infarction Patients Direct to the Catheterization Lab

**DOI:** 10.7759/cureus.74674

**Published:** 2024-11-28

**Authors:** Matthew J Levy, Asa Margolis, Victoria Collins, Daniela Krahe, Eric Garfinkel, J. Lee Jenkins, Becca Scharf, Patricia Pugh, Eric Schwartz, Zachary Tillett, Peter Johnston

**Affiliations:** 1 Department of Emergency Medicine, Johns Hopkins University School of Medicine, Baltimore, USA; 2 Department of Fire and Rescue Services, Office of the Chief Medical Officer, Howard County Maryland, Mariottsville, USA; 3 Department of Fire and Rescue Services, Office of the Chief Medical Officer, Howard County Maryland, Marriottsville, USA; 4 Department of Fire and Rescue Services, Howard County, Marriottsville, USA; 5 Department of Emergency Medicine, Johns Hopkins Howard County Medical Center, Columbia, USA; 6 Department of Cardiology, Johns Hopkins Howard County Medical Center, Columbia, USA; 7 Department of Cardiology, Johns Hopkins University School of Medicine, Baltimore, USA

**Keywords:** cath lab activation, emergency medical services, myocardial infarction, stemi, stemi alert

## Abstract

Background

Rapid treatment of ST-elevation myocardial infarction (STEMI) patients with primary percutaneous coronary intervention (PCI) significantly reduces morbidity and mortality rates. Recent studies emphasize the importance of reducing total ischemic time, making first-medical-contact-to-balloon (FMCTB) time a key performance indicator. To improve FMCTB times in patients brought to the Emergency Department (ED) by Emergency Medical Services (EMS), we implemented a "Direct to Lab" (DTL) workflow during the following conditions: weekday daytime hours, when the lab is fully staffed, and for hemodynamically stable STEMI patients presenting via EMS.

Methods

We performed a pre/post analysis following the implementation of a pilot workflow for EMS STEMI patients to be rapidly triaged to the cardiac catheterization lab as compared to those patients who underwent the standard workflow before program implementation at a 225-bed community hospital in a suburban setting in Maryland, USA. The hospital’s STEMI database was queried from 2/1/2021 through 3/1/2024, including all EMS STEMI alert activations during the study period. Cases were excluded if the patient arrived after program operating hours, declined PCI, or if clinical circumstances (such as cardiac arrest or the need for other resuscitative or diagnostic interventions) necessitated additional ED stabilization before PCI.

Results

A total of 30 patients met the inclusion criteria. The analysis revealed significantly reduced ED, door-to-balloon (DTB), and FMCTB times for patients under the "Direct to Lab" workflow, including a total ED time of 8.4 minutes faster, an average DTB time of 19.6 minutes faster, and an average FMCTB time of 24.3 minutes faster than those triaged via the standard workflow. Complication rates were similar among both groups. The most common reason that stable patients were not taken directly to the lab was the need for further clinical evaluation before cardiac catheterization or the lab not being immediately available.

Conclusion

In this pilot single-center analysis, STEMI patients who were expeditiously triaged "Direct to Lab" experienced significantly lower total ED, DTB, and FMCTB times with no difference in procedural complications. This study highlights the patient-centered benefits of a robust collaboration between EMS, ED, and Interventional Cardiology teams.

## Introduction

As the most severe and time-sensitive form of coronary artery disease, ST-elevation myocardial infarction (STEMI) makes up 38% of acute coronary syndrome patients who present to the Emergency Department (ED) [1]. Emergency medical care for acute STEMIs necessitates immediate percutaneous coronary intervention (aka “primary PCI”), which has been shown to reduce morbidity and mortality significantly and have a lower complication rate compared to thrombolysis [2,3]. However, the success of primary PCI is contingent on the timely initiation of the initial medical contact [4-6]. Recognizing STEMI as a time-sensitive condition underscores the critical role of expedited PCI, which aims to promptly restore perfusion to ischemic cardiac tissue, minimizing cardiac tissue death and improving patient outcomes. Traditional practices involve EMS transporting STEMI patients to the ED for evaluation and potential stabilization before proceeding to the cardiac catheterization laboratory (CCL). Several studies have shown that pre-hospital ECG and CCL activation before patient arrival in the ED are associated with reduced intervention times [7-11].

Efforts to improve STEMI treatment times have historically focused on metrics that begin once the patient arrives at the hospital, using door-to-balloon (DTB) time as the primary metric [12]. More recently, studies have demonstrated that mortality and infarct size correlate more strongly with total ischemic time than DTB [6]. Consequently, achieving a first-medical-contact-to-balloon (FMCTB) time of less than 90 minutes has emerged as a key performance indicator (KPI) for STEMI management. Opportunity exists to improve the efficiency of getting STEMI patients to the CCL while optimizing human and systems-based factors [11]. Emergency Medical Services (EMS) systems thus focus on rapid STEMI diagnosis and timely communication with hospital personnel to facilitate primary PCI [6]. Hospitals, in turn, continually refine workflows to minimize the time elapsed from STEMI onset. Interventions that bypass the ED, delivering patients directly to the CCL, are associated with significant reductions in DTB and FMCTB times [10-13].

In March 2022, Johns Hopkins Howard County Medical Center (HCMC), a 225-bed community hospital in a large suburban area, implemented a STEMI patient workflow termed "Direct to Lab" (DTL), for patients who were identified as a prehospital STEMI alert and had Cath lab activation by Howard County paramedics. During weekday daytime hours (7 am to 4 pm), hemodynamically stable STEMI patients diagnosed on field ECG and transported to the HCMC ED by the Howard County Department of Fire and Rescue Services (HCDFRS) were taken directly to the CCL by HCDFRS after hospital registration and a brief screening exam by an emergency department attending physician to rule out the need for further evaluation or stabilization before the CCL. To facilitate this process, the ED attending physician and triage nurse met the EMS crew at the ED ambulance entrance and rapidly screened the patient, keeping the patient on the ambulance cot, as the patient was quickly registered in the electronic medical record system. If the patient did not require any other immediate stabilization, the ED staff would then accompany EMS to take the patient to the cath lab, where a handoff of care occurs.

This study compared selected KPIs (specifically DTB and FMCTB times) of EMS patients with STEMI who underwent DTL triage in comparison to patients triaged under the traditional workflow during the preceding calendar year who presented during the same hours (weekdays 7 am to 4 pm) and met the same clinical criteria applied to DTL patients. We secondarily sought to compare complication rates between these groups and to determine why some patients who presented with STEMI during weekday and daytime hours after the DTL program was begun were not managed through the DTL workflow.

## Materials and methods

The study environment, Howard County, Maryland, is a suburban county between Baltimore, Maryland, and Washington, DC. The county is 251,000 square miles and has a population of 336,000 [14].HCDFRS is the sole provider of EMS and advanced life support care to Howard County and responds to approximately 30,000 calls for services annually, of which approximately 80% result in transports being taken to HCMC. All HCDFRS STEMI patients have the prehospital ECG transmitted to the HCMC ED before ED arrival. EMS ECGs are reviewed in real-time by a board-certified/board-eligible attending emergency physician and the on-call interventional cardiologist, who collaborate in the decision to activate the CCL.

HCMC, a member of Johns Hopkins Medicine, is a 225-bed comprehensive acute care facility with a 36-bed emergency department and an annual volume of 77,000 patients. From 2019 to 2022, the CCL performed an average of 84 primary PCI cases annually. HCMC and HCDFRS leadership team members participate in monthly CCL case conferences, including detailed clinical program performance reviews. During one of these monthly meetings, committee members discussed the concept that led to developing the DTL pilot program, and the project was planned. Additionally, HCMC interventional cardiologists routinely participate in HCDFRS EMS clinician continuing education for all HCDFRS paramedics. These educational sessions include case reviews, program performance, and best practices. These sessions were used to educate the paramedics about the DTL program.

We conducted a pre/post-implementation study of the HCMC CCL STEMI activation program's performance database, including time metrics and clinical outcomes related to the implementation of the DTL pilot program. This database includes all cases where an activation request is sent from the ED attending physician. The study period included data from the 12 months before the pilot program’s implementation and was analyzed from 2/1/2021 through 3/1/2024. All EMS STEMI alert activations during the DTL program's operational hours, weekdays, 7 am to 4 pm, were included. Cases were excluded if the patient arrived outside the program’s operating hours, declined PCI, or had clinical circumstances such as cardiac arrest or other resuscitative or diagnostic interventions that necessitated additional ED stabilization before PCI.

A descriptive summary was then created (Microsoft Excel, Microsoft Corporation, Redmond, WA, USA) consisting of the following variables: date/time of the STEMI alert, daytime hours vs. after hours, age/gender/race of patient, whether the patient was taken “DTL,” pertinent medical information (chief complaint, presence of condition requiring resuscitation), prehospital arrival times, time of first ECG diagnostic of STEMI, STEMI notification time, CCL activation time, FMCTB time), intervention performed, and the occurrence of any complications. Summary descriptive statistics and a Wilcoxon signed-rank test for statistical significance were then performed (R Core Team, Vienna, Austria). This study was determined to be exempt by the Johns Hopkins School of Medicine Institutional Review Board, study ID: IRB00431411.

## Results

**Figure 1:**
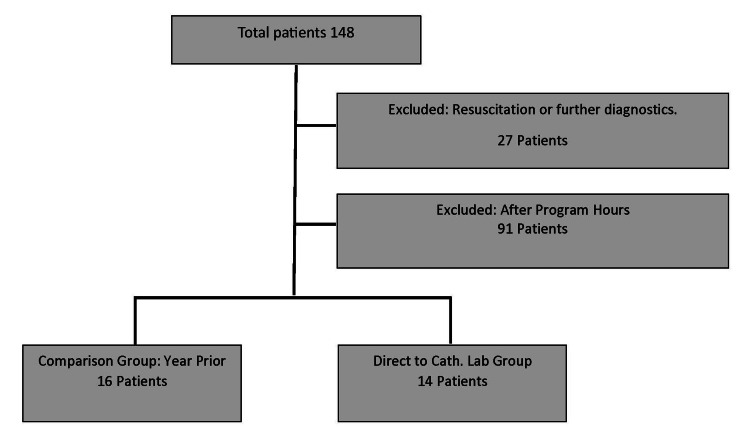
Study population

A total of 148 patients arrived at HCMC as EMS STEMI alert notifications during the study period. Of these patients, 27 were excluded due to the requirement for ongoing resuscitation or diagnostic intervention, and 91 were excluded because they arrived outside the DTL program’s operating hours. The analysis focused on the remaining 30 patients who arrived during weekday daytime hours. Among these 30 patients, 14 (46%) were taken DTL under the new workflow. These 14 patients were compared to the 16 patients from the 12 months preceding the program who similarly presented during daytime hours not requiring resuscitation or further diagnosis, which served as the comparison group (Figure 1). 

The 14 cases (one female, 13 male, average age 54.8 years (range: 36-73)) that were taken DTL had a mean total ED time of 10.7 (range: 3-22); median 9.5 minutes; a mean door-to-balloon time of 35.9 (22-56), median 33.5 minutes; and a mean first-medical-contact-to-balloon time of 59.6 (44-84), median 59.0 minutes. In comparison, in the 12 months preceding the program (aka the comparison group), there were a total of 16 patients (4 female, 12 male, average age 64.9 years (range: 50-83)) who presented during daytime hours had a mean total ED time of 19.1 (range: 7-35), median 17.5 minutes; a mean DTB time of 55.5 (range: 28-105), median 51 minutes; and a mean FMCTB time of 83.9 (range: 56-145), median 79.0 minutes. On average, DTL patients experienced a total ED time of 8.4 minutes faster, an average DTB time of 19.6 minutes faster, and an average FMCTB time of 24.3 minutes faster than those triaged via the standard workflow. This translates to average reductions of 44% in total ED time, 35.3% in DTB time, and 28.9% in FMCTB time, respectively. A Wilcoxon signed-rank test indicated that median total ED time (Z=2.79, P<0.01), DTB time (Z=2.91, P<0.01), and FMC2D time (Z=3.28, P<0.001), ranks for the direct to lab group were statistically significantly lower than the comparison group. These results are further illustrated in Table 1.

**Table 1:** Descriptive statistics and Wilcoxon signed-rank test comparison between “direct to lab” and standard workflow patients (statistical significance p<0.01) DTB: door-to-balloon; FMC2B: first-medical-contact-to-balloon

Category	Comparison Group	Direct to Lab	Percent Reduction	z-Score	p-Values
n	16	14			
Gender					
Male	12	13			
Female	4	1			
Age					
Mean (CI)	64.9 (60.3 – 69.5)	54.8 (47.0 – 62.5)			
Median	65.0	58.0			
Range	50 - 83	36 - 73			
ED Time (mins)					
Mean (CI)	19.1 (14.7 – 23.6)	10.7 (7.4 – 14.0)	44.0%		
Median	17.5	9.5	45.7%	2.79	0.0053
Range	7 – 35	3 – 22			
DTB Time (mins)					
Mean (CI)	55.5 (44.0 – 67.0)	35.9 (30.5 – 41.4)	35.3%		
Median	51.0	33.5	34.3%	2.91	0.0036
Range	28 – 105	22 - 56			
FMC2B Time (mins)					
Mean (CI)	83.9 (71.1 – 96.7)	59.6 (65.7 – 53.6)	28.9%		
Median	79.0	59.0	25.3%	3.28	0.0010
Range	56 – 145	44 - 84			
Complications	4 (25%)	4 (29%)		-0.22	0.825

The most common reasons for not going directly to the lab after the program was initiated included four patients who required additional diagnostic evaluation (usually CT imaging) but did not require active resuscitation, three cases in which the lab was in use for another case (3), a communications barrier delaying the assessment and consent process (3), and lab team member availability (2). Among the 14 cases taken DTL, 4 (29%) were noted to have complications, with 3 instances having arrhythmia and 1 stent thrombosis. Conversely, of the 16 cases handled via standard workflow, 4 patients (25%) experienced complications that included arrhythmia, agitation, pseudoaneurysm, and cardiac arrest. A Fisher's exact test demonstrated an odds ratio of 0.83 (p=1.0) and a Z-score of approximately -0.22 (p=0.825), indicating no statistically significant difference in the complication rates between the two groups.

## Discussion

A well-established and robust body of literature has demonstrated the importance of early coronary revascularization for improving outcomes in patients experiencing STEMI. The time-critical nature of these emergencies has informed the development of systems of care intended for rapid detection, intervention, and reperfusion of cardiac tissue, thus mitigating infarct size and secondary complications. This study showed the impact of an optimized cath lab activation process for EMS STEMI patients and its ability to improve FMCTB times, thus decreasing the patient's total ischemic time.

With guidelines reinforcing its importance, efforts to decrease FMCTB times may seem intuitive and straightforward [15]. However, these practices must be implemented locally and may vary based on resource access and availability. Additionally, guidelines and best practice recommendations do not account for individual hospitals' more minor yet salient nuances, including but not limited to variations in staffing, registration systems, cath lab activation processes/procedures, and stakeholder engagement. Operational issues may impede efficiency at multiple points in the STEMI activation timeline. For example, in more rural areas, the total ambulance transport time to a hospital with PCI capability is a significant factor, regardless of the time to CCL activation [7,16].

Clinical considerations, including patient acuity, need for active resuscitation, and EMS clinician ECG interpretation proficiency, may affect prehospital activation of the CCL [17,18]. Once a STEMI patient arrives in the ED, FMCTB time may increase if hospital staff hesitate to activate the CCL based on the EMS-transmitted ECGs [16,19]. Additional delays may occur if interventional cardiology is not consulted immediately or available [19]. For example, among the 47 patients who arrived after hours (after 4 pm through before 7 am) when the DTL workflow was not in use, the mean DTB time was 67.8 minutes. The mean FMCTB time was 93.9 minutes for patients who arrived after hours compared to 55.5 (DTL group) and 83.9 minutes (comparison group). Although excluded from this analysis, this suggests that the legacy workflow achieves similar KPI independent of the time of day.

The successful implementation of this program relied on educating and garnering support from team members from across the STEMI system of care. Strategic planning for this program took over six months and encompassed team meetings, updating policy, tabletop exercises, mock patient scenarios, stakeholder engagement, and training efforts. Program awareness was messaged through written communications, face-to-face shift briefings, and impromptu training sessions. Case feedback was provided during comprehensive STEMI work group meetings. Relevant case feedback was also shared with team members to help facilitate process adjustments and workflow optimization. We also diligently strived to promptly deliver positive feedback to clinical team members (including EMS clinicians).

Several factors may limit the generalizability of these results. This study was a retrospective, pre/post-implementation study and not a hypothesis-testing randomized trial. Despite efforts to ensure adequate controls, as a retrospective study, the risk of bias must be acknowledged. This study setting was a single center with a single EMS system, and HCMC and HCDFRS have a decades-long relationship in emergency care collaboration. This study analyzed time variables beginning with the patient's ED arrival, as the ED direct-to-lab process was the only workflow variable changed for this project. It did not evaluate other prehospital time variables, such as differences in EMS transport, ECG acquisition times, field activation times, and their relationship to observed times. To ensure staff buy-in and education, centers looking to adopt similar processes should anticipate adequate resource investment. Expanding programs like DTL depends upon the immediate availability of interventional cardiology staff and resources. Therefore, the DTL program was deliberately limited to weekday daytime hours when the interventional cardiology team members were onsite. 

## Conclusions

An expedited direct-to-cardiac catheterization lab triage process was associated with significant time savings for hemodynamically stable EMS STEMI patients and was not associated with statistically significant higher complications. Interdisciplinary quality improvement processes are essential in implementing innovations to advance emergency cardiac care and optimizing treatment processes. As STEMI care systems continue to evolve, initiatives like this serve as models for enhancing efficiency and quality of care, ultimately benefiting patients.
